# A Narrative Review on the Sperm Selection Methods in Assisted Reproductive Technology: Out with the New, the Old Is Better?

**DOI:** 10.3390/jcm14041066

**Published:** 2025-02-07

**Authors:** Angeliki Tiptiri-Kourpeti, Byron Asimakopoulos, Nikolaos Nikolettos

**Affiliations:** 1 Genesis Athens—Thrace Medically Assisted Reproduction Unit, Apalos, 68100 Alexandroupolis, Greece; 2 Laboratory of Physiology, Faculty of Medicine, Democritus University of Thrace, 68100 Alexandroupolis, Greece; basima@med.duth.gr; 3 Obstetric and Gynecologic Clinic, Medical School, Democritus University of Thrace, 68100 Alexandroupolis, Greece; nnikolet@med.duth.gr

**Keywords:** swim-up, DGC, MACS, microfluidics, sperm sorting, assisted reproduction

## Abstract

**Background:** Male infertility, accounting for nearly half of infertility cases worldwide, has spurred significant research into its causes, diagnosis, and treatment strategies. Genetic abnormalities, social causes, environmental exposures, lifestyle, and further health conditions are key contributors. **Methods:** Essential to improving the outcomes of ART is, among other things, the selection of high-quality sperm, which requires methods that assess sperm motility, morphology, DNA integrity, and oxidative stress levels. **Results:** Traditional techniques such as semen analysis, swim-up, and density gradient centrifugation (DGC) are still widely used, but there is ongoing discussion regarding the limitations in detecting DNA damage and oxidative stress. Advanced methods like magnetic-activated cell sorting (MACS) and microfluidic sorting have emerged as more precise tools for selecting sperm with better genetic integrity, although they face challenges in terms of their standardization, cost, and clinical adoption. Emerging technologies such as artificial intelligence (AI) and Raman spectroscopy offer the potential for more automated, accurate sperm selection, minimizing human error and variability. However, the integration of these methods into clinical practice requires further validation through large-scale studies, including assessments of their long-term safety and cost-effectiveness. **Conclusions:** Future research should focus on refining sperm selection techniques, tailoring them to personalized infertility approaches, and addressing the gaps in the evidence to improve ART outcomes and patient care.

## 1. Introduction

Male infertility accounts for almost half of the burden of infertility in couples from various parts of the globe and affects a significant percentage of the world’s population. This condition has influenced much interest in research on male fertility factors, diagnostic approaches, and intervention strategies [1,2]. Among various causes, genetic background and social and environmental actors have been identified as the main reasons for the prevalence of male infertility [3,4,5]. Unhealthy dietary patterns affect sperm count, concentration, and motility and increase DNA fragmentation [6,7]. Malignant diseases, especially testicular tumors and Hodgkin’s lymphoma, have been reported to lower semen concentrations and other parameters depending on treatment [8,9]. Virus infections, including infection during the recent SARS-CoV-2 pandemic, are also a potential fertility threat [10,11,12]. HPV semen infection, beyond the oncogenic risks involved, seems to be related to unexplained infertility and low sperm motility [13,14,15]. HPV, Chlamydia trachomatis, and AAV are considered risk factors for infertility, while HBV and HPV have been reported to affect assisted reproduction outcomes [14,16]. Ureoplasma and Mycoplasma subspecies induce urogenital inflammation and decrease sperm quality [17,18].

These parameters are hard to identify in many cases since male factor infertility is usually asymptomatic. As such, most cases can only be diagnosed when couples seek reproductive support on account of difficulty conceiving [19,20,21]. To many such couples, assisted reproductive technology (ART) has offered promising solutions to their infertility problems, especially methods such as in vitro fertilization (IVF) and intracytoplasmic sperm injection, with ICSI being exceptionally helpful in cases where infertility is due to severe male factor infertility [22,23,24]. According to the sixth edition of WHO’s laboratory manual for the examination and processing of human semen, the basic parameters for healthy sperm are progressive motility > 30%, total sperm number > 39 × 10^6^, a sperm concentration > 16 × 10^6^, vitality > 54%, and normal sperm morphology > 4%. Traditionally, motility is measured in wet specimens 30 min from ejaculation under a microscope. Sperm concentrations are measured preferably using a Neubauer or else a Makler chamber. Vitality is evaluated through eosin/nigrosin staining or the HOS test and morphology mainly using Papanicolaou or Diff-Quik stain. All of these parameters can be estimated using a computer-assisted sperm analysis—CASA (9789240030787-eng.pdf (https://iris.who.int/bitstream/handle/10665/343208/9789240030787-eng.pdf?sequence=1, access date: 30 January 2025)).

In an attempt to optimize ART outcomes, the selection of high-quality sperm is obviously an important aspect. The techniques of sperm selection aim to isolate the healthiest sperm based on their motility, morphology, and genetic integrity parameters in order to increase fertilization rates and optimize embryo development [1,25,26]. Traditional semen analysis assesses sperm in terms of its motility, concentration, vitality, and morphology, and these parameters remain critical to the initial diagnostic evaluations [27,28]. However, a conventional semen analysis cannot reflect the finer details of sperm quality, especially with respect to DNA integrity and other molecular biomarkers, recently recognized as influential for fertilization success and embryo quality [29,30].

One major area of inquiry into male factor infertility is that of oxidative stress and how it affects sperm quality. Oxidative stress can be measured using direct and indirect laboratory techniques. The most commonly used indicators are oxidation–reduction potential (MiOXSYS System) and total antioxidant capacity (OxiSperm, Halotech; CANros, Candore) [31]. OS is a result of the imbalance between ROS and antioxidants. Reactive oxygen species produce oxidative stress and result in cellular damage, as the body’s antioxidant capacity is insufficient. Although these levels of ROS are naturally occurring, they can increase due to environmental influences like infection or lifestyle factors, including smoking or poor diet [32,33,34]. Consequently, essential sperm parameters and hence fertilization capability are affected [35,36,37]. The reactive oxygen species (ROS) produced by the leukocytes or granulocytes negatively impact human spermatozoa by significantly impairing their motility and morphology. Thus, a reduction in sperm hyperactivation and the ability to penetrate the oocyte is observed [38]. Interestingly, seminal plasma from infertile men contains higher levels of nitric oxide (NO) compared to that from fertile men, which hampers sperm capacitation and binding with oocytes [39,40]. Since the state of oxidative stress affects sperm function and integrity directly, sperm selection techniques may become of primary importance in attempts to improve the outcomes of assisted reproductive technology [41,42]. Techniques like magnetic-activated cell sorting (MACS), which eliminate apoptotic sperm with deteriorated membranes, have been promising in reducing ROS-induced impairment and thereby improving pregnancy rates with the use of ART, as evidenced by Esteves et al. [43] and Dirican et al. [44].

High DFI values are associated with poor outcomes, even from ICSI, as reported by Zhao et al. [45] and Zini and Agarwal [46]. Various DNA fragmentation measurement methods have been employed, including Sperm Chromatin Structure Assay (SCSA), Single-Cell Gel Electrophoresis (SCGE/COMET), Deoxynucleotidyl Transferase-Mediated dUTP Nick End Labeling (TUNEL), and Sperm Chromatin Dispersion (SCD). Fertility clinics mainly use and evaluate SCD (halo) tests that do not require a flow cytometer and use light microscopy [47]. DNA fragmentation, as quantified through the DNA fragmentation index (DFI), represents another critical biomarker in assessing sperm quality for ART. A high DFI has consistently been associated with reduced pregnancy rates and an increased risk of miscarriage due to the importance of DNA integrity in embryo development [22,48,49]. Age is the most common cause of high DNA fragmentation [50]. Semen with abnormal sperm parameters has a higher possibility of an elevated DFI [51]. A recent metanalysis identified varicocele, impaired glucose tolerance, testicular tumors, smoking, pollution, and advanced male age as basic factors increasing sperm DNA fragmentation [52]. Advanced testing methods, such as the TUNEL assay and Sperm Chromatin Structure Assay (SCSA), have made it possible over time to detect sperm with impaired DNA integrity more precisely and systematically, thereby helping in the more effective selection of sperm [53,54,55].

All of this emergent awareness has inspired a plea for improved techniques for sperm selection based on the underlying problems in terms of quality issues, hence affording much more refined selections than those allowed using conventional assessments [56]. Indeed, novel approaches to sperm selection, such as microfluidic sorting, which selects out sperm with a low DFI, have demonstrated the potential to improve the success rates of ART by isolating sperm with superior genetic integrity [45,57,58]. Microfluidics itself is an emerging technique that effectively models the natural processes of sperm selection by using a physical barrier through which only the most motile of sperm can pass. It is also expected to enhance clinical outcomes since it selects sperm with a lower DFI and a better morphology [25,59,60]. Proteomics and molecular profiling provide further insight into sperm selection and the outcomes of ART. Proteomics can be defined as a subdiscipline that studies the protein composition of sperm cells, which can identify unique biomarkers associated with fertility potential, hence allowing for more personalized treatment options for ART [53,61,62]. By using proteomics, various other proteins responsible for sperm motility, vitality, and DNA integrity can be identified, which probably further presents additional selection methods. For instance, the acrosome reaction is related to proteins enabling sperm to penetrate the egg; thus, successful fertilization relies on these proteins, which become markers that have to be present in functional sperm selection [63,64,65]. This has been informed by advances in epigenetic profiling, which looks at modifications of DNA without actually changing the DNA sequence and demonstrates environmental and lifestyle-related influences on sperm health, including potential molecular markers of sperm quality assessment [66,67,68].

While techniques such as MACS, microfluidic sorting, and advanced proteomic profiling have had promising results, due to the absence of standardization for these methodologies, substantially high costs, and a lack of large studies providing related evidence, they are not used commonly in clinics [59,69,70]. In this regard, it is essential for there to be full evidence-based guidelines stating the recommendations for various sperm selection techniques for different etiologies of male infertility. In the future, research should be directed towards clinical trials that will establish the efficacy of new emerging techniques for sperm selection, identify indications for their use, and finally assess their cost-effectiveness to facilitate their wider application in ART [23,57,71]. Thus, the following review will critically assess the current literature reporting on sperm selection techniques for use in ART, both traditional and emerging ones, and identify their strengths and weaknesses while identifying future directions. We synthesize evidence for the impact of these methods on the outcomes of ART to provide an overview of the field, including constructive recommendations for further research and clinical practice. The ensuing review thus sets the future course of study in focusing on the gaps, working out better protocols, and ultimately increasing the success rates for couples trying ART.

## 2. Sperm Selection Techniques

**Figure 1:**
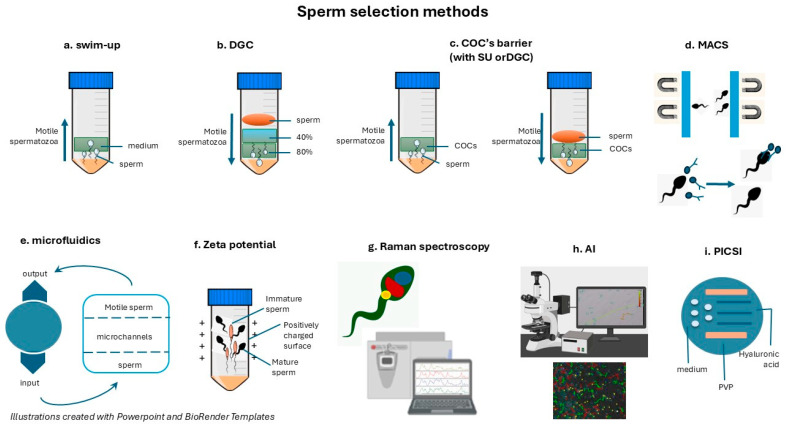
Sperm selection methods—there are conventional and advanced sperm sorting techniques. The most widely used or promising for integration into clinical practice are illustrated.

Conventional and advanced sperm selection techniques have changed assisted reproduction step by step by improving the outcomes and thus providing solutions for male infertility factors conventionally untreatable using the standard diagnostic or therapeutic measures. In general, the aims of effective sperm selection methodologies are to isolate the sperm cells with the best motility, morphology, and genetic integrity; this improves fertilization success rates and embryo quality in ART [29,69]. These techniques range from the conventional swim-up, density gradient centrifugation (DGC), COC barrier selection, and HOS tests to more advanced magnetic-activated cell sorting (MACS), microfluidics, and other newer techniques (Figure 1). Each of these has various advantages and limitations, making any particular method preferable depending on the underlying factor of infertility and the specific needs of ART.

### 2.1. Conventional Sperm Selection Methods

#### 2.1.1. The Swim-Up Technique

Swim-up is the oldest and one of the easiest and most cost-effective sperm selection methods; it finds wide usage due to the simplicity of the approach, and it is very effective in selecting motile sperm (>90%). In this method, a sperm sample is layered underneath a medium; hence, motile sperm swim upwards into the medium, leaving the immotile or less viable cells behind [41,72]. Because motility is a criterion for natural selection, swim-up enriches the sample with active motile sperm, which correlates with an enhanced fertilizing potential [70]. Therefore, this technique has the advantage of being able to isolate sperm with minimal DNA fragmentation since highly motile sperm bear lower levels of DNA damage [73,74]. While effective, the swim-up method possesses some drawback and limitations in cases of sperm with poor motility, as seen in severe male factor infertility. Because only a fraction of the motile sperm swims up into the medium, yields are usually low, and this technique is not usually applied in oligospermic or asthenozoospermic individuals [69]. Some researchers have suggested that pellet swim-up can be used to reduce the cell stress caused by the multiple rounds of centrifugation in other methods and improve the recovery rate of mature spermatozoa [75]. Nevertheless, in patients with severe male factor infertility, pelleted spermatozoa have been documented to create increased ROS and thus reduce motility [76]. Studies have demonstrated that swim-up, while adequate for IUI, is limited in yield and hence restricted in its utilization for intensive ART processes such as ICSI or IVF, where higher-quality sperm samples may be in demand [77,78].

#### 2.1.2. Density Gradient Centrifugation (DGC)

DGC is a widely used sperm selection technique, and based on the cell density, it separates healthy and motile sperm from debris, leukocytes, and morphologically abnormal spermatozoa [73,79]. Centrifugation renders the sample layer over a colloidal silica gradient, thus allowing for high-quality motile sperm to be collected at the bottom layer. Indeed, various studies have shown that DGC effectively selects sperm with high motility and intact DNA to improve the ART outcomes compared to those when using unprocessed semen [32,59]. Of particular note, DGC has been quite successful in IVF and ICSI settings, where a high yield of motile and morphologically normal sperm is crucial for successful fertilization and embryo quality. However, there are some disadvantages to DGC, including the oxidative stress that can be generated during the centrifugation procedure. The generation of oxidative stress is also well documented to result in DNA fragmentation in spermatozoa, which will influence fertilization and subsequent embryo development [3]. In mainly targeting motility and morphology but not DNA fragmentation, DGC is not effective for the patients mentioned above because the DNA fragmentation is >30% [74]. Given these caveats, DGC has remained a mainstay in ART, although it is usually combined with advanced selection methods to further ensure the integrity of the selected sperm.

#### 2.1.3. Comparison of Swim-Up and DGC

Swim-up and DGC represent two efficient options for motility- and morphology-based sperm selection. However, swim-up and DGC significantly differ in their application and limitations. Swim-up results in minimal generation of oxidative stress and represents the treatment of choice when motility is sufficient; however, the low yield associated with it limits its utility in ICSI and IVF [72,73]. Conversely, DGC allows for favorable advantages for procedures that require a higher volume of motile sperm, such as IVF, although it can introduce oxidative stress, affecting DNA integrity in vulnerable individuals [77,80]. Comparative studies show that the swim-up method results in greater sperm viability and motility, but DGC involves greater total motile sperm counts [81]. However, the clinical pregnancy rates (CPRs) and live birth rates (LBRs) are similar between these methods [76]. Hence, the conventional methods are usually selected according to the specific needs of the ART and the sperm quality profile of the patient.

#### 2.1.4. “Physiological” ICSI (COC Barriers)

“Physiological” ICSI stands for a biologically representative method of sperm selection using a cumulus oophorus complex. The cumulus cells serve as a barrier allowing the spermatozoa to reach the zona pellucida of the oocyte and achieve fertilization. Spermatozoa that can accomplish this passage have better structural, functional, and metabolic characteristics. Sperm are tasked with passing through the cumulus complex alone or, usually, in combination with the help of conventional selection methods. Carell et al. studied sperm morphology and capacitation during IVF and found that within the cumulus, the spermatozoa had a statistically improved morphology, and most tapered sperm were excluded. Moreover, the acrosome reaction statistically increased [82]. In accordance with these findings, COC-selected spermatozoa present better chromatin condensation and a higher zona-binding capacity [83,84]. Glass capillaries have also been used with cumulus cells for sperm selection. Sperm function analyses showed that compared with the use of spermatozoa incubated in medium only, those exposed to COCs were more likely to have a normal morphology, be capacitated and acrosome-reacted, and have a distinct motility pattern and better zona-binding capacity [85]. “Physiologically” selected spermatozoa have also been documented to have significantly less DNA fragmentation compared to that in sperm samples that are selected using conventional methods alone [86]. ICSI, even though it did not demonstrate a significantly higher cleavage rate, showed improved blastocyst formation rates and scores [87]. Sperm isolated after DGC using a COC have shown higher mean fertilization rates, embryo quality, and chemical pregnancy, clinical pregnancy, and ongoing pregnancy rates in couples with male factor infertility undergoing ICSI [88]. Swim-up in combination with the use of a COC recovered high-motility and high-quality spermatozoa with enhanced mitochondrial functionality and reduced sperm DNA damage [89]. In the absence of advanced and expensive sperm selection methods, the use of a cumulus oophorus complex presents a viable alternative. This approach can assist in ICSI, where the selected spermatozoa are often immotile due to their low motility or retrieval from the testes [84].

#### 2.1.5. Brief Overview of Other Conventional Methods

There are additional conventional techniques that have been employed over the years but are used only for specific infertility conditions or are not used anymore due to the existence of more advanced techniques with better ART outcomes. These techniques include HOSTs (hypo-osmotic tests) and LAISS (Laser-Assisted Immotile Sperm Selection) that select viable immotile spermatozoa. The HOS test is simple and economical and is based on the swelling of a sperm cell’s tail when it is incubated in hypo-osmotic solution [90]. The HOST results in low fertilization rates when the hypo-osmotic incubation lasts for more than 30 min, and it is not recommended for low volumes of semen [91]. LAISS, on the other hand, is based on the curling of the flagellum when it is hit by a laser and is recommended for primary ciliary dyskinesia or Kartagener’s syndrome, but it has a high cost, and the possibility of membrane rupture and ROS production is high [92]. Annexin V is also employed to recognize apoptotic spermatozoa. Annexin V is conjugated with a fluorophore and indicates spermatozoa with a higher fertilization potential. Moreover, it is documented that annexin-selected spermatozoa have a superior morphology [93]. Very promising agents are methylxanthines. These are used to promote motility in sperm. Pentoxiffyline triggers sperm motility, even in testicular samples, and provides vital sperm for ICSI [94]. Theophylline has the same stimulatory effect as pentoxiffyline. Studies have shown that sperm incubated with theophylline achieve better fertilization, blastulation, and implantation rates and CPRs [95]. Due to their deleterious effect on oocytes, methylxanthines are advised to only be used on totally immotile spermatozoa [96]. Morphological abnormalities have been addressed using IMSI (intracytoplasmic morphologically selected sperm injection), which selects spermatozoa based on their ultrastructure. IMSI is documented to increase blastocyst and implantation rates in male factor infertility but has conflicting ART results [97]. It is expensive and time-intensive and requires extensive training [98,99]. Another fast and safe method is horizontal sperm migration which involves the preparation of a modified ICSI plate that includes a greater number of culture medium drops than that used in horizontal swim-up bridges [100]. Even though this technique reduces ROS, it is not advised for oligospermic semen samples, and the clinical results are conflicting. There are more substances used specifically to select better spermatozoa, e.g., Myoinositol [101,102] for OAT samples and ATP/MgSO_4_ for immotile spermatozoa, but these have been characterized better as activating agents than selection techniques [97].

### 2.2. Advanced Sperm Selection Techniques

#### 2.2.1. Magnetic-Activated Cell Sorting (MACS)

Magnetic-activated cell sorting (MACS) is the most recent and sophisticated technique for the sorting of non-apoptotic sperm, though the separation of apoptotic cells is based on the expression of phosphatidylserine—a marker for apoptosis. For MACS, magnetic microbeads are conjugated with annexin V, which means annexin V only binds to apoptotic sperm [103]. Similarly, upon their exposure to a magnetic field, human vital cells are isolated. So far, this technique has been efficient in cases with increased oxidative stress and DNA fragmentation, as it discards harmed or dying sperm while enriching samples with DNA-intact, viable sperm [54], thus providing a comprehensive evaluation of the sperm’s molecular properties [104]. There is conflicting evidence regarding MACS. Although there is a trend of it improving ART outcomes by enhancing fertilization rates and achieving reduced-duration, cost-effective treatments and a better embryo quality in cases of severe male factor infertility [42,46], studies also underline that MACS shows minimal statistically significant changes in CPRs and LBRs [93,105]. A recent study by Mantravadi and Rao compared the outcomes using MACS and TESA in patients with >30% sperm DNA fragmentation. No significant differences were detected in blastocyst formation, miscarriage rates, or LBRs, although the MACS group had a higher implantation rate [106]. Recent studies have investigated the combination of MACS with DGC to further improve the selection efficacy since DGC isolates motile sperm, while MACS ensures that their DNA is intact. Combining these two may ensure a synergistic effect for high-risk cases [107]. Although studies agree that these techniques combined can lead to the best results as far as ART is concerned, mainly in cases with high DNA fragmentation or with recurring failures of ART treatment, a recent systematic review did not find any statistically significant change in the overall ability for filtered sperm to result in pregnancy relative to that with the traditional methods [108]. Sperm sorting using MACS has significant differences compared to the conventional methods, mainly in terms of high SDF levels. Considering its cost-effectiveness, MACS in combination with SU or DGC does not achieve a significant overall improvement in ART and causes the loss of progressive motile spermatozoa. Moreover, it is expensive and not widely available, particularly in resource-limited areas. It is not recommended as a routine technique, but it could be useful for overcoming the miscarriage rates in autologous ICSI and achieving higher pregnancy rates and LBRs [109,110]. It would be valuable for subsequent research to focus on whether and how MACS actually improves pregnancy rates and reduces miscarriage rates [111].

#### 2.2.2. Microfluidic Sperm Sorting

Microfluidic sperm sorting is an emerging technology that selects sperm through the reconstitution of the natural mechanisms of selection by microfluidic channels, letting the most motile sperm pass through while subjecting them to the minimum mechanical and oxidative stress [25]. Microfluidics has become an emerging tool for processing relatively low-volume samples, and this technology is a hot topic in infertility diagnosis and treatment [112]. Generally, microfluidic devices are considered cost-effective, especially in male infertility. Nevertheless, the device itself increases the cost compared to that with the classic selection methods. The device fabrication and the equipment needed contribute to the overall cost. This may restrain their wide availability and adoption, particularly in regions with limited resources [104]. Unlike conventional techniques, microfluidics has no need for centrifugation; hence, as a result, it can lower the risk of DNA damage drastically [57,113]. This method therefore offers an advantage in the settings of ART, as highly motile, morphologically entitled, and DNA-intact sperm samples will be produced, which is an essential condition for successful IVF and ICSI results [70]. Indeed, microfluidic sorting has been shown to be very helpful in cases of male infertility with high levels of DNA fragmentation because it enriches samples with sperm that possess lower fragmentation indices and a better morphology compared to that in other methods [25,114]. Banti et al. have documented that the use of sperm sorting microfluidic chips can improve blastocyst formation, utilization, and euploidy rates following ICSI in comparison to those with the DGC method. Moreover, no significant association was found with the presence or absence of male factor infertility [115]. Researchers have reported promising results from microfluidics—better fertilization rates, improved embryo development, and superior live birth outcomes in the ART setting—thus establishing the former as a low-stress and highly effective technique for sperm selection [69]. Further investigation will extend the technology needed for clinical applications. Studies should focus on improving the efficacy, reproducibility, and ease of the common use of these devices [116].

#### 2.2.3. Zeta Potential Selection

Zeta potential selection in sperm sorting is a novel method involving the use of an electrical charge on sperm to distinguish mature, high-quality cells from immature or damaged ones. This is evidence for proper epididymal maturation and spermatogenesis in the testes [117]. Zeta potential is based on the fact that mature spermatozoa have a negative membrane electrical charge (−16 mV to −22 mV) [118] that decreases with capacitation or exposure to follicular fluid and neuraminidase from the uterus. This electrokinetic potential, which represents the potential in the slip plane between the sperm membrane and its surroundings, has been named the zeta potential [119]. A decreased telomere length in spermatozoa is associated with male infertility. Measuring the zeta potential can select more spermatozoa with intact chromatin compared to using DGC alone [120]. Mature and better-quality sperm are highly charged and immobilized while lesser charged sperm and debris are washed away. Attachment activates sperm metabolism without a premature acrosome reaction. The selection of the zeta potential for research purposes has been shown to decrease DNA fragmentation and produce a high-quality sperm population, which is promising in various ART applications [70,121]. Zeta potential selection is less clinically utilized than MACS or microfluidics because of the specialized equipment requirements. Still, zeta analyzers are fast and simple and are considered relatively cost-efficient [122]. When microfluidics is not an option, using the zeta potential over MACS is proposed due to its simplicity and cost-effectiveness [123]. Early studies have reported improved outcomes with respect to fertilization rates and embryo quality when zeta-selected sperm are used. These results indicate that zeta potential could be one of the selection factors in specific cases of sperm selection. Forthcoming research should involve larger studies to show how zeta potential correlates with fertilization success and embryo development. Moreover, it would be useful to investigate how zeta potential can be integrated with other sperm selection methods, like microfluidics, to enhance ART outcomes [124].

#### 2.2.4. Hyaluronic Acid (HA)-Binding Assay (PICSI)

The HA-Binding Assay selects for the DNA integrity and functional maturity of sperm through the natural selection of HA by mature sperm. Sperm with defective chromatin are not able to digest HA effectively. This technique involves using HA-coated plates, to which mature, viable sperm selectively bind, thereby mimicking the natural selection processes in the female reproductive tract [32,78]. Indeed, to this day, most studies have shown that HA-binding sperm are usually those with low DNA fragmentation and hence can serve as ideal ones for ICSI, for which DNA integrity is considered one of the important factors regarding the quality of the embryos and the respective pregnancy outcomes [42,53]. The HA-Binding Assay has been noted to improve the outcomes of ART, mainly by increasing embryo quality and pregnancy rates. The effective binding of HA in ICSI procedures for the non-invasive isolation of mature sperm supplements the treatment and embryo development in cases of male infertility related to high DNA fragmentation [70,72]. A meta-analysis documented that PICSI has similar LBRs to ICSI, although a reduction in miscarriage rates is observed [125]. PICSI is actually an affordable and widely used technique in clinical routine, but many concerns have arisen because of its weakness in enhancing LBRs. It may offer a benefit in male infertility cases with high DNA fragmentation and/or oxidative stress [126]. In the future, additional studies should investigate how PICSI compares to traditional ICSI in diverse patient groups and its cost-effectiveness in improving ART [127].

#### 2.2.5. Raman Spectroscopy and Artificial Intelligence (AI)

Raman spectroscopy and AI are considered novel technologies in sperm selection, as they can provide non-invasive evaluations and accurate sorting. Raman spectroscopy has become a very useful tool for obtaining details about the molecular structure and properties of sperm through the analysis of vibrational transitions [128]. By employing Raman spectroscopy, one can study sperm at a molecular level without destroying their viability based on the detection of specific markers related to DNA integrity and metabolic profiles that enhance ART selection [64,129]. Spermatozoa have a unique, simple structure and are easily recognizable. Although some studies have not detected the same spectroscopy peaks, preventing definite conclusions about the relevant components, there is an important peak at 1092 cm^−1^ produced by the PO_4_ DNA backbone. Sperm DNA damage can be evaluated using this peak [130]. In this way, Raman spectroscopy can identify and evaluate with relative accuracy sex chromosomes, aneuploidies, oxidative DNA damage, and, generally, structural and functional-integrity-related aspects of spermatozoa [131,132,133]. Raman spectroscopy is expensive due to the equipment required and the specialized expertise in handling and interpreting the data. However, once the system is established, it offers the advantage of non-invasive, real-time analysis [134]. Raman spectroscopy is mainly limited to research laboratories and is not yet widely accessible in fertility clinics [135]. Further investigation could improve its sensitivity, cost, and applicability in clinical settings. Validation and optimization of Raman analysis against more traditional methods with larger samples will ensure consistent reproducible results.

AI is powered by machine learning algorithms, where improvements in the selection processes are automated to pick out subtle indications of sperm quality that might be overlooked in a manual selection process. Artificial intelligence approaches are based on computational imaging processes following specific software protocols. In most cases, microscopy is combined with holography, and precise information about the optical path of the specimen can be excluded [136]. One of the most significant tools of predictive value is validation of the dataset used. The clinical outcomes are better fitted if an external, multicentered, international dataset is used based on clinical, hormonal, and semen data [137]. The cost of AI systems largely depends on the scale of their deployment and the level of sophistication of the algorithms used. AI-based sperm analyses can be cost-effective long-term and reduce the costs associated with morphological analyses by automating image analysis, which requires expert training and equipment [138]. Their large-scale adoption is constrained by regulatory hurdles, the high initial costs, and the need for infrastructure upgrades. Interestingly, artificial intelligence sperm selection systems are becoming more available in fertility clinics, though they are not yet universally used (Kharazmi et al., 2022). Kharazmi et al. highlighted that it is necessary to improve the data collection, train AI systems, and ensure the AI models are effective in diverse populations [139].

Indeed, Raman spectroscopy and AI remain experimental and require further research to clinically validate them; however, they seem to hold promise for improving the outcomes of ART. These technologies have the potential to provide further support to non-destructive, precision-based selection processes in the future of ART [67].

### 2.3. Comparative Analysis of the Techniques

Each sperm selection method has its particular advantages and limitations, and its applications are highly contextual under ART. Conventional sperm selection methods are well established in assisted reproduction clinics. They rely on centrifugation separation and sperm’s physical swimming ability. These techniques emphasize distinguishing motility and morphology and have limited precision in selecting sperm with specific characteristics. The ART outcomes are rarely well predicted, but the use of conventional methods with rather lower DNA fragmentation seems to be successful [140].

The conventional approaches, swim-up and DGC, are proficient in the isolation of sperm bearing good motility and a normal morphology since these parameters are involved in fertilization success. Swim-up is indicated in relatively high-motility conditions, giving sperm with minimal DNA fragmentation. However, DGC separates sperm based on density and thus gives higher yields of motile sperm suitable for intensive ART procedures like IVF, and as such, it has a broader applicability [43,78]. However, these two techniques are documented not to be suitable for individuals with a high degree of DNA fragmentation or oxidative stress since neither of these types of prepared sperm involve direct selection concerning DNA integrity, hence limiting the outcomes in high-risk cases [3,74]. The most critical issue here is that DGC and swim-up are often inadequate on their own for producing the desired results and need supplementation with advanced techniques in order to achieve better outcomes, especially in patients whose sperm quality is poor.

More sophisticated sperm separation methods explore its functional characteristics and prioritize the role of DNA integrity, capacitation, and gene expression patterns. Advanced techniques such as MACS, microfluidic sorting, and the HA-Binding Assay have increased the efficiency of the selection techniques to improve the selection of sperm with intact DNA, either targeting apoptotic sperm or cells with compromised integrity. For example, MACS has been useful in a number of clinical cases for reducing the DNA fragmentation and oxidative stress in sperm samples. This is because MACS selectively removes apoptotic sperm with damaged cell membranes. Indeed, evidence has shown that MACS brings added value to DGC in the improvement of clinical ART outcomes, and more so in cases of high oxidative stress [42,46]. Accordingly, MACS may be preferred in patients with repeated implantation failure or poor embryo development since it consistently provides lower DNA fragmentation levels in sperm, indicative of higher pregnancy and live birth rates [107].

Another advanced technique that has recently gained a lot of attention is microfluidic sperm sorting due to the high integrity of the DNA of the sperm selected due to its simulation of the natural processes of sperm selection in microchannels. In contrast to centrifugation-based techniques, microfluidics minimizes the oxidative stress and mechanical strain, thereby reducing DNA fragmentation significantly [57,113]. Microfluidic selection usually offers better-quality embryos and reduces the miscarriage rates in IVF and ICSI through the selection of sperm with lower DNA fragmentation indices and a better morphology. By nature, microfluidics are non-invasive and hence used in ART cases in which the minimum stress should fall on the sperm [114]. This is one of the major reasons why it is used for complete male infertility cases and repeated ART failure [25]. Both the HA-Binding Assay and zeta potential selection represent new approaches to the selection of sperm in relation to its maturity and integrity, with each being based on different physiological properties. The HA-Binding Assay isolates mature sperm capable of binding to HA, which correlates with DNA integrity and reduced fragmentation. This method has given promising results in intracytoplasmic sperm injection, where the selected sperm improve the embryo quality and clinical pregnancy rates [70]. In turn, zeta potential selection separates mature and high-quality sperm from immature or damaged ones according to their electrical charge characteristics. Though it is less ubiquitously applied than other advanced methods, it has shown efficacy in reducing DNA fragmentation and yielding sperm populations more likely to support successful ART outcomes [121].

New technologies, including Raman spectroscopy and AI-assisted selection, will bring in and shape a new generation of precision-based sperm selection. Raman spectroscopy can be used to carry out non-invasive analyses of the biochemical composition of human sperm, thereby allowing clinicians to evaluate the DNA integrity and metabolic markers important for fertilization without compromising cell viability [64]. Backed by advanced imaging, AI demonstrates promise in fully automating the identification of high-quality sperm through subtle morphological and functional markers that enable faster, more accurate selection for ART [129]. Though the clinical application of these methods is still in its relative infancy, their principles represent advancements toward individualized treatment strategies—ranging a further step into a sophisticated, data-driven approach to sperm selection. In Table 1, the advantages and disadvantages of the previously discussed sperm sorting methods used in assisted reproductive technologies (ARTs) are summarized.

To sum up, advanced sperm sorting techniques enhance the prediction of ART outcomes, basically through understanding and taking into account the functional attributes of the exact spermatozoa. Success rates seem to be improved, mainly due to a better embryo quality, but their potential clinical cost-effectiveness is reduced for economic reasons and due to the maintenance durations and their universal availability [108,141].

**Table 1:** Principles, advantages, and limitations of the conventional and advanced sperm sorting techniques discussed. Prices mentioned may vary in different regions. Green stands for an advantage, yellow for a moderate effect, and red for a disadvantage.

Method	Principle	Operator	Reagents-Equipment	Cost-Effectivenss	Invasiveness-Time	Advantages-Limitations	References
Swim-up	Separates motile sperm in culture medium. Spermatozoa swim upwords in the supernatant	Easy to perform but requires some essential expertise	Widely available	Cost-effective (~30 euro/cycle)	Non invasive, time efficient	Selects motile sperm with fair DNA integrity—effective for IUI and normal/ fair sperm parameters	[123,142,143,144,145]
						Sperm with DNA fragmentation remains—ineffective in low motility/ concentration cases	
Density Gradient Centrifugation	Seperates sperm through a density gradient by centrifugation	Easy to perform but requires some essential expertise	Widely available	Cost-effective (~50 euro/cycle)	Non invasive, time efficient	Motile sperm with good morphology, removes debris. Improves fertilization rates in oligo/ astheno-zoospermia	[3,32,79,143,146,147,148]
						Sperm with high DFI may not benefit due to centrifugation, ineffective in severe infertility/ immotile spermatozoa	
COC’s barrier	Uses cumulus oophorus complex to mimic natural sperm selection	Easy to perform but requires preparation	In fresh cycles available COCs	Cost-effective (~70 euro/cycle)	Non invasive, time efficient	High motility and morphology sperm, capacitated. Mainly used in combination with SU or DGC to improve blastulation	[86,87,88,89]
			Limited COC availability in cycles with cryopreserved oocytes			Ineffective in low concentration cases	
MACS	Sperm selection based on the exclusion of apoptotic cells with magnetic microbeads coated with antibodies	Requires special training but can be automated	Not universally available, specialized reagents and equipment. Technical considerations for clinical use (calibration - quality guarantee)	High cost (~180 euro/cycle), plus high equipment and functional cost	Non invasive, time efficient	Sperm with intact membranes and low DNA fragmentation. Fertilization and pregnancy rates imrpovent in OS/ repeated implantation failure	[106,109,149,150,151,152,153]
						Basically used to severe asthenospermia with high DFI and absolute immotility	
Microfluidics	Sperm selection through microchannels. Mimics natural selection	Easy to perform but requires some essential expertise	Widely available	Moderate cost (~100 euro/cycle)	Non invasive, time efficient	Sperm with high motility and good morphology. Requires low semen volume. Good for high DFI and OAT to improve CPR, LBR	[121,154,155,156,157,158,159,160]
						Not suitable for severe oligo or/ and asthenozoospermia and absolute immotility	
Zeta Potential Selection	Viable sperm isolation with electrical charge	Easy to perform but requires training	Need for special equipment	High cost (~160 euro/cycle), plus equipment cost	Non invasive, moderate duration	Viable sperm isolation. Reduced sperm loss. Improvement of CPR, LBR	[123,156,161,162,163,164]
						Not suitable for severe oligo or/ and asthenozoospermia and absolute immotility. Early clinical adoption	
Raman Spectroscopy	Molecular fingerprinting of sperm	Requires expert training	Need for special equipme	High cost (~300 euro/cycle), plus high equipment cost	Non invasive, time efficient	Detailed molecular analysis of sperm - genetic profile	[130,131,135,165,166,167,168]
						Limited clinical validation - ethical considerations. Mostly experimental technique	
AI (Artificial Inteligence)	Analyze sperm parameters with algorithms	Automated. Requires extensive data for training	Need for special equipment. Requires IT infrastructure	High cost (~500 euro/data setup), plus high equipment cost	Non invasive, time efficient	High motility and morphology sperm, good DNA integrity. Reduces human error	[128,139,169,170,171]
						User evaluation is advised. Used mostly for analysis, rather sorting in clinical use	
PICSI	Sperm selection with hyaluronan binding. Mimics natural fertilization	Easy to perform but requires ICSI training	Widely available, but needs ICSI equipment	Moderate cost (~100 euro/cycle), ICSI station considered installed	Non invasive, time efficient (combining ICSI duration)	Ideal for OAT, high DFI, high misscarriage rate. Improves blastulation, CPR	[32,125,172,173,174,175,176,177]
						Not applicable to all infertility types	

## 3. Discussion

The traditional methods currently employed in sperm selection for ART indeed have advanced the field, but at the same time, they are burdened with a host of challenges. For instance, the conventional swim-up and DGC methods remain crucial, though they are generally limited in targeting key parameters such as DNA integrity, one of the most important factors in fertilization success. We must take into account that the traditional techniques are based on centrifugation-assisted separation. In ART, a potential source of ROS in the culture media arises during semen preparation. This can lead to ROS production by immature sperm, often triggered by centrifugation in the absence of antioxidant-rich seminal plasma or due to factors such as leukocyte contamination, oxygen exposure, light, the culture medium conditions, cryopreservation, and thawing [178]. During ART procedures, the gametes are manipulated and prepared for fertilization, leading to variations in the ROS sources between conventional IVF and ICSI. The centrifugation process, routinely used in sperm preparation, has been shown to elevate ROS levels and induce oxidative stress in the spermatozoa. It has been observed that repeated centrifugation cycles significantly increased ROS production in human sperm, with the duration of centrifugation playing a more critical role than its intensity. This process also contributed to greater DNA fragmentation, adversely affecting ART outcomes [179]. Moreover, ROS possibly increase the DFI following sperm apoptosis [180]. A high DFI, even in normozoospermia, enhances the likelihood of miscarriage [181] and negatively influences pregnancy rates, embryo quality [182,183] and blastulation formation and scores [184].

Advanced techniques like MACS, microfluidics, and the HA-Binding Assay have overcome some of these deficiencies, but inconsistencies in the results remain a challenge. Most of the variability arises through procedural differences, skills, and the specific characteristics applying to each individual case of infertility [185]. For example, the effectiveness of MACS or microfluidic sorting depends on both the expertise of the operator in applying the technique and their ability to obtain viable, DNA-intact sperm. The standardization of these new techniques, along with best practices, may help reduce such variability, especially when such protocols are introduced for specific infertility etiologies, such as high DNA fragmentation and oxidative stress [54]. While much has been promised by the current sperm selection techniques, all of these techniques remain manual and may increase the chances of human error, besides being highly operator-dependent. This dependency suggests the need for approaches that can reduce the variability and optimize the outcomes through automation, thus potentially enhancing ART success rates. Given such challenges, the research is increasingly oriented toward the integration of emerging technologies into ART. For instance, AI and algorithms in machine learning are at the dawn of changing sperm selection by automating analyses of sperm morphology and motility, even down to the biochemical markers associated with DNA integrity [186] (Zhou et al., 2020). AI-based systems can evaluate very minute parameters that may be overlooked during manual selection, hence increasing the accuracy in selecting high-quality sperm. Concomitantly, non-invasive spectroscopic methods, such as Raman spectroscopy, provide promising perspectives concerning assessing sperm’s biochemical properties without cell destruction and selecting sperm with the ideal metabolic and genetic profiles. It has been revealed that these technologies could make the selection more effective and reduce operator-related variability considerably [64].

Beyond the performance of the sperm selection methods in terms of the sperm characteristics per se, it is also highly valuable to consider and assess the particular ART outcomes. We have already discussed how the conventional techniques are the most widely used for sperm preparation regardless of the assisted reproduction method used. However, the existing correlations between ART outcomes are inadequate. A comparison of the first autologous cycles that used DGC or swim-up showed no significant differences in the fertilization rates, embryo quality, blastocyst formation rates, or cumulative LBRs and LBRs per transfer following conventional IVF or ICSI. Interestingly, sperm of low quality that was selected using DGC achieved a statistically higher fertilization rate [187].

**Figure 2:**
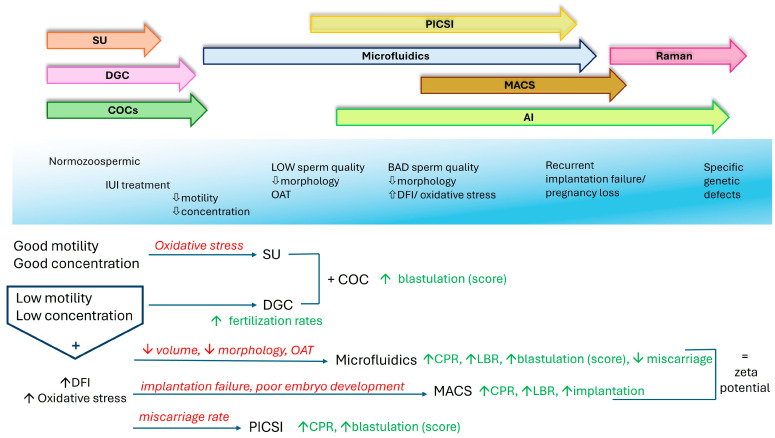
Sperm selection method decision: The selection of the preferable technique for each incident varies and is not always clear. SU is mainly used when the sperm parameters are not strongly affected. Otherwise, it is better to use DGC. In samples with a reduced sperm motility and/or concentration, the classic methods are best, combined with COC sorting. A reduced normal sperm morphology leads to the use of more sophisticated techniques, starting with microfluidics and PICSI, mainly due to their comparative availability and lower cost. As additional damaging parameters increase, such as the DFI and oxidative stress, other techniques are used as supplements, such as MACS, although there are not enough clinical data. In fact, in the absence of these advanced methods, it is suggested that the zeta potential can perform equally. AI has up until now been used primarily for functional analyses rather than sorting, although AI results are interpreted in the decision on which sperm selection method is more suitable. Raman spectroscopy needs to be evaluated for clinical use in order to be used for specific metabolic and genetic profiles.

Conventional methods have been compared to advanced techniques in order to assess whether the new approaches are superior in terms of predicting ART outcomes and success. New technologies and machine learning algorithms provide excellent opportunities to select the most promising sperm candidates [188]. Sperm selection using microfluidic devices rather than swim-up in ICSI cycles for male factor infertility showed no differences in the fertilization rate and embryo quality but resulted in statistically higher pregnancy rates, CPRs, and LBRs [160]. DGC and microfluidic sperm selection was compared for couples with repeated implantation failure and high DNA fragmentation. Even though microfluidics did not achieve higher fertilization rates, euploidy rates, clinical miscarriage rates, or LBRs, it resulted in a higher number of top-quality blastocysts [189]. Other groups have also documented similar results on euploidy rates [190]. Microfluidic devices have been documented to significantly improve blastocyst development rates in donor oocytes and cryopreserved sperm [191] and improve blastocyst formation, utilization, and euploidy rates compared to conventional methods [115,192,193]. In cases with elevated sperm DNA fragmentation, while sperm selection using TESE produced slightly higher blastocyst formation rates, MACS resulted in higher implantation rates [106]. Moreover, a tendency to improve the LBR in the first embryo transfer and the cumulative LBR was documented for MACS over DGC and swim-up combined [149]. The mean fertilization, day 3 embryo quality, and clinical pregnancy and implantation rates were higher when the sperm selection was performed using MACS and DGC combined than DGC alone [194]. Hozyen’s group studied couples with abnormal SDF undergoing ICSI and sorted the sperm using DGC, TESA, PICSI, or MACS. Mainly, PICSI and MACS showed better implantation rates, CPRs, and ongoing pregnancy rates and, along with DGC, had higher blastulation rates and high blastocyst quality [177]. MACS seems to be better when the females are younger than 30 years, while PICSI is preferred in older females [195], but overall, PICSI improves the embryological and clinical parameters of ART outcomes [196,197,198,199,200]. Still, some articles have reported that only sperm with low SDF produced a considerably higher blastulation rate using PICSI or that it made no difference at all [201,202]. Considering the above knowledge, we created a basic graph to present our hypothesis on the use of sperm selection methods in the near future (Figure 2). Despite the conflicting research results, we created a flowchart that depicts the resulting reasoning behind the choice of each method.

One question is apparent. Are the new-age procedures better than the old-school techniques? This review tries to fathom the results and comprehend whether the advanced sperm selection methods provide real assistance or will remain an experimental add-on to in vitro fertilization. Do the biological fertility markers used based on advanced computation-related methods reliably and accurately predict successful fertilization? How do age, hormonal and social profiles, and genetic predispositions influence each selecting technique’s efficacy? Are there long-term implications for the health of the offspring?

There is a real need for clinical studies assessing the long-term outcomes in offspring conceived using advanced sperm selection techniques. While most of the available information is concentrated on ART’s short-term outcomes—namely fertilization and pregnancy results—the developmental, health, and epigenetic effects of certain sperm selection techniques must be understood to establish these methods’ long-term safety and efficiency [203]. Attention to the study of a population over time may show, by means of DNA integrity, whether methods like MACS or microfluidic sorting have a positive effect in decreasing genetic problems or developmental disorders in offspring conceived through ART. Leveraging ensemble machine learning models using traditional sperm quality assessments is required. For instance, MACS and microfluidic devices show promise in cases with high DNA fragmentation, while the conventional methods may be sufficient for sperm with minimal abnormalities. Molecular diagnostics can be combined with artificial intelligence to enable clinicians to predict the most suitable sperm selection method. In this way, ART can be used through a more meticulous and personalized route, eventually offering better outcomes for combating infertility [204]. To validate this hypothesis, further large-scale, longitudinal research on the use of sperm selection, including its impact on the genetic and epigenetic components of embryos, will be useful. Designing personalized fertility treatments can take the precision in ART to the next level, which may include customized strategies for couples facing specific infertility situations.

All of these technologies, which allow for more precise and efficient sperm selection, hold significant promise for improving ART outcomes. However, they also present ethical dilemmas related to human enhancement, discrimination, and the commodification of human life. One of the most significant ethical concerns is the potential use of eugenics in selecting traits that align with social or cultural preferences. Technologies like Raman spectroscopy enable the identification of not only motile, capacitated spermatozoa but also the absence of certain genetic characteristics. Reproductive technologies that facilitate genetic selection may exacerbate societal inequalities by reinforcing discriminatory practices based on genetic traits. Spermatozoa with certain genetic markers could be used preferably and lead to the exclusion of minority genetic backgrounds [205]. Furthermore, Spar et al. raise the issue of the commodification of reproductive materials. Sperm selection could be driven by economic reasons rather than fertility issues [206]. Since the gamete market is legally regulated in most regions and is considerably expanded, the ability to choose specific genetic traits could reduce the perceived value of children to mere commodities. Sperm selection based, for example, on physical appearance or athleticism could further entrench social inequalities and lead to further marginalization [205]. The impact on parental and child autonomy is discussed as well. Specific sperm selection could lead to parents imposing unrealistic aspirations on their children and may influence the expectations placed on a child from birth [207]. Selecting for specific genetic characteristics could narrow a child’s future autonomy and identity [208]. Ethical frameworks must be developed to balance the potential benefits of these technologies with the protection of human autonomy and diversity.

With personalized approaches, sperm genetic and molecular profiling can be employed in tailoring the selection methods to specific infertility profiles. Expanded success, while minimizing the risks related to ART, is possible. The integration of these new technologies with the existing practices in ART will therefore play an important role in the future in overcoming many of the conventional pitfalls in sperm selection. Larger studies are also needed in order to provide standardization for a variety of clinical scenarios so that these new tools can be used consistently and thus benefit more patients. In addition, the assessment of the cost-effectiveness of such technologies in real clinical settings will also be important to widely facilitate their dissemination. Guidelines and criteria for the success benchmarks regarding advanced sperm selection must be developed based on an evidence-based framework that asserts their efficacy and safety to both practitioners and patients.

## 4. Conclusions

This narrative review presents a practical and comparative synthesis of the literature to declare that sperm selection techniques form an integral part of assisted reproductive technologies, each with an advantage between the conventional and advanced techniques dictating an appropriate match with different infertility factors. The conventional techniques of swim-up and DGC laid the basis for selection based on motility and morphology, whereas MACS, microfluidics, and HA-binding were developed to focus further on DNA integrity and biochemical profiles, enhancing the results in terms of the ART outcomes for complex cases. In particular, the emerging technologies of AI and spectroscopic analysis will further revolutionize sperm selection by bringing automation and precision, which may wipe out some of the present drawbacks in ART practices—that is, the variability and skill dependency. For such technologies to be fully integrated into the standard protocols of ART, more extensive research on their long-term safety, efficacy, and cost-effectiveness are considered essential. Major development and refinement of various sperm selection techniques, through the use of technological innovations amply validated by confirmatory empirical evidence, will result in huge improvements in ART outcomes, increasing the chances of success for childless couples and creating healthier future generations.

The conclusions of this review provide a general background by focusing on the selection of the most effective sperm sorting method and demand a nuanced, individualized approach that incorporates the newest available ART practices. Emerging evidence shows that no single sperm selection technique is universally superior; rather, the ideal technique mainly depends on individual factors. So, the old techniques are good enough, but in severe male infertility and in combined and unexplained infertility, there is a gap waiting to be bridged. The broader application of non-invasive techniques will enhance safety and accessibility, ensuring that personalized fertility care becomes a global standard. More definitive guidelines could be established through systematic reviews with meta-analyses in order to address and summarize all relevant individual studies and make the evidence accessible. This field can continue to evolve with the particular needs of each patient, and a new era of reproductive medicine—both precise and patient-centered—can be fostered.

## Abbreviations

MACS	Magnetic-Assisted Cell Sorting
DGC	Density Gravity Centrifugation
SU	Swim-Up
ART	Assisted Reproduction Technology
AI	Artificial Intelligence
HPV	Human Papilloma Virus
HBV	Hepatitis B Virus
ICSI	Intracytoplasmic Sperm Injection
IVF	In Vitro Fertilization
HOS test/HOST	Hypo-Osmotic Swelling Test
WHO	World Health Organization
CASA	Computer-Assisted Sperm Analysis
ROS	Reactive Oxygen Species
NO	Nitric Oxide
DFI	DNA Fragmentation Index
SCD	Sperm Chromatin Dispersion
SCSA	Sperm Chromatin Structure Assay
OS	Oxidative Stress
TUNEL	Terminal Deoxynucleotidyl Transferase dUTP Nick End Labeling
COC	Cumulus Oophorus Complex
PICSI	Physiological Intracytoplasmic Sperm Injection
IUI	Intrauterine Insemination
CPR	Clinical Pregnancy Rate
LBR	Live Birth Rate
IMSI	Intracytoplasmic Morphologically Selected Sperm Injection
OAT	Oligoasthenoteratozoospermia
TESA	Testicular Sperm Aspiration
HA	Hyaluronic Acid
SDF	Sperm DNA Fragmentation
